# Author Correction: Quantifying hierarchy and dynamics in US faculty hiring and retention

**DOI:** 10.1038/s41586-023-06379-9

**Published:** 2023-07-05

**Authors:** K. Hunter Wapman, Sam Zhang, Aaron Clauset, Daniel B. Larremore

**Affiliations:** 1 Department of Computer Science, University of Colorado Boulder, Boulder, CO USA; 2 Department of Applied Mathematics, University of Colorado Boulder, Boulder, CO USA; 3 BioFrontiers Institute, University of Colorado Boulder, Boulder, CO USA; 4 Santa Fe Institute, Santa Fe, NM USA

**Keywords:** Careers, Institutions

Correction to: *Nature* 10.1038/s41586-022-05222-x Published online 21 September 2022

When building on this research, we found a discrepancy between (i) how new hires were identified in our code and (ii) how new hires were described as being identified in the Methods section of the paper. We have therefore updated the text in both the HTML and PDF versions of the paper to read “we labelled faculty as new hires if they earned their degree within 4 years of their first recorded employment as faculty.” This accurately reflects the analyses performed. Further detail may be found at https://larremorelab.github.io/us-faculty/. We sincerely regret the error.

