# Supplementary material for: Quantifying hierarchy and dynamics in US faculty hiring and retention
Source: Nature. 2022 Sep 21;610(7930):120–7. doi: 10.1038/s41586-022-05222-x (PMC9534765; doi:10.1038/s41586-022-05222-x)
Supplement: Supplementary file 1 — Reporting Summary [file 41586_2022_5222_MOESM1_ESM.pdf]

nature portfolio

Corresponding author(s): Daniel Larremore

Last updated by author(s): July 29, 2022

Reporting Summary

Nature Portfolio wishes to improve the reproducibility of the work that we publish. This form provides structure for consistency and transparency in reporting. For further information on Nature Portfolio policies, see our Editorial Policies and the Editorial Policy Checklist.

Statistics

For all statistical analyses, confirm that the following items are present in the figure legend, table legend, main text, or Methods section.

n/a	Confirmed	
☐	☒	The exact sample size (*n*) for each experimental group/condition, given as a discrete number and unit of measurement
☒	☐	A statement on whether measurements were taken from distinct samples or whether the same sample was measured repeatedly
☐	☒	The statistical test(s) used AND whether they are one- or two-sided<br>*Only common tests should be described solely by name; describe more complex techniques in the Methods section.*
☐	☒	A description of all covariates tested
☐	☒	A description of any assumptions or corrections, such as tests of normality and adjustment for multiple comparisons
☐	☒	A full description of the statistical parameters including central tendency (e.g. means) or other basic estimates (e.g. regression coefficient) AND variation (e.g. standard deviation) or associated estimates of uncertainty (e.g. confidence intervals)
☐	☒	For null hypothesis testing, the test statistic (e.g. F, t, r) with confidence intervals, effect sizes, degrees of freedom and P value noted<br>*Give P values as exact values whenever suitable.*
☒	☐	For Bayesian analysis, information on the choice of priors and Markov chain Monte Carlo settings
☒	☐	For hierarchical and complex designs, identification of the appropriate level for tests and full reporting of outcomes
☐	☒	Estimates of effect sizes (e.g. Cohen's *d*, Pearson's *r*), indicating how they were calculated

Our web collection on statistics for biologists contains articles on many of the points above.

Software and code

Policy information about availability of computer code

Data collection	See Data and Approach for information on original data obtained from AARC. See Methods for information on additional information on how universities were hand-annotated with country labels.
Data analysis	Open-source code related to this study is available at https://doi.org/10.5281/zenodo.6941612.

For manuscripts utilizing custom algorithms or software that are central to the research but not yet described in published literature, software must be made available to editors and reviewers. We strongly encourage code deposition in a community repository (e.g. GitHub). See the Nature Portfolio guidelines for submitting code & software for further information.

Data

Policy information about availability of data

All manuscripts must include a data availability statement. This statement should provide the following information, where applicable:

- Accession codes, unique identifiers, or web links for publicly available datasets
- A description of any restrictions on data availability
- For clinical datasets or third party data, please ensure that the statement adheres to our policy

All network data associated with this study, and all data contained in Extended Data tables are freely available in machine-readable format at https://doi.org/10.5281/zenodo.6941651. Explorable visualizations of faculty hiring networks are available at larremorelab.github.io/us-faculty/, and for university ranks, at larremorelab.github.io/us-institutions/.

March 2021

Human research participants

Policy information about studies involving human research participants and Sex and Gender in Research.

Reporting on sex and gender	This manuscript discusses gender, but not sex. As stated in Methods, we used self-identified genders when possible, and machine-annotated genders otherwise:<br><br>Self-identified gender annotations were provided for 6% of faculty in the unprocessed dataset. In order to annotate the remaining faculty with gender estimates, we used a two-step process based on first and last name. First, complete names were passed to two offline dictionaries: a hand-annotated list of faculty employed at Business, Computer Science, and History departments (corresponding to data used in Ref. [27]), and the open-source python package gender-guesser [58]. Both dictionaries responded with one of the following classifications: female, male, or unable to classify. Second, for the cases where the dictionaries either disagreed or agreed but were unable to assign a gender to the name, we queried Ethnea [59] and used the gender they assigned the name (if any). Using this approach we were able to annotate 85% of faculty with man or woman labels. Faculty whose names could not be associated with a gender were excluded from analyses of gender but included in other analyses. This methodology associates names with binary (man/woman) labels because of technical limitations inherent to name-based gendering methodologies, but we recognize that gender is nonbinary. The use of these binary gender labels is not intended to reinforce the gender binary.
Population characteristics	Our analysis examines tenured or tenure-track faculty employed in the years spanning 2011 and 2020 at 368 PhD-granting universities in the U.S., each of whom is annotated by their doctoral institution, year of doctorate, faculty rank, and faculty gender. To be included in our analysis, a professor must be a member of the tenured or tenure-track faculty at a department that appears in the majority of sampled years, which yields n = 295, 089 faculty in 10,612 departments.
Recruitment	This dataset resulted from cleaning and preprocessing a larger U.S. faculty census obtained under a Data Use Agreement with the Academic Analytics Research Center (AARC). This dataset spanned all tenure-track and tenured faculty at U.S. PhD-granting institutions, between 2011-2020.
Ethics oversight	After consultation with the University of Colorado Boulder IRB, protocol submission and approval was deemed unnecessary for the present study, due to is secondary use of publicly available data.

Note that full information on the approval of the study protocol must also be provided in the manuscript.

Field-specific reporting

Please select the one below that is the best fit for your research. If you are not sure, read the appropriate sections before making your selection.

☐ Life sciences ☒ Behavioural & social sciences ☐ Ecological, evolutionary & environmental sciences

For a reference copy of the document with all sections, see nature.com/documents/nr-reporting-summary-flat.pdf

Behavioural & social sciences study design

All studies must disclose on these points even when the disclosure is negative.

Study description	This study quantitatively analyzes patterns found in qualitative data, namely the records of individual tenure-track or tenured faculty at U.S. PhD-granting institutions between 2011-2020. Namely, we analyze PhD institution, current department and institution, faculty rank, and gender. By observing new entrants to the dataset over time, or departures over time, we also analyze hiring and attrition of said U.S. tenured or tenure-track faculty.
Research sample	All tenure-track and tenured faculty at U.S. PhD-granting institutions, except for faculty of Law and Medical schools. This sample represents a ten-year annual census of these faculty and is representative due to its complete coverage; this is not a random subsample of the population being studied.
Sampling strategy	Census sampling was used by the original data providers (AARC). In some cases, data were reported to the AARC directly by institutions themselves. In all other cases, the AARC (or their affiliates) collected faculty rosters and doctoral degree information from public-facing university webpages, annually. The data provided to the research team spanned only 2011-2020, though the sampling strategy has been in use by the AARC or their affiliates for years prior to our sample frame.
Data collection	Our data resulted from cleaning and preprocessing the larger academic census dataset obtained under a Data Use Agreement with the Academic Analytics Research Center (AARC), who collected the original dataset as described above. Please see Methods for detailed descriptions of the nine key cleaning steps, and two key annotation steps that were used prior to the manuscript's analyses.
Timing	2011 to 2020
Data exclusions	A complete description of data exclusions and cleaning — and which data were excluded or included for each analysis — is included in Methods.

Non-participation	Not applicable.
Randomization	Not applicable.

Reporting for specific materials, systems and methods

We require information from authors about some types of materials, experimental systems and methods used in many studies. Here, indicate whether each material, system or method listed is relevant to your study. If you are not sure if a list item applies to your research, read the appropriate section before selecting a response.

Materials & experimental systems

n/a	Involved in the study
☒	☐ Antibodies
☒	☐ Eukaryotic cell lines
☒	☐ Palaeontology and archaeology
☒	☐ Animals and other organisms
☒	☐ Clinical data
☒	☐ Dual use research of concern

Methods

n/a	Involved in the study
☒	☐ ChIP-seq
☒	☐ Flow cytometry
☒	☐ MRI-based neuroimaging
